# Reductions in angiotensin II type 2 receptor-mediated vasodilation contribute to increased angiotensin II vasoconstrictor sensitivity in women with preeclampsia history

**DOI:** 10.1042/CS20245238

**Published:** 2025-06-11

**Authors:** Kelsey S. Schwartz, Nathan Campbell, Diana I. Jalal, Anna E. Stanhewicz

**Affiliations:** 1 Department of Health and Human Physiology, The University of Iowa, Iowa City, Iowa, U.S.A.; 2 Department of Internal Medicine, Carver College of Medicine, Iowa City, Iowa, U.S.A.; 3 Department of Pharmacology & Toxicology, University of Mississippi Medical Center, Jackson, Mississippi, U.S.A.; 4 The Iowa City VA HCS, Iowa City, lowa, U.S.A.

**Keywords:** angiotensin II, microvascular, preeclampsia, postpartum, vasoconstriction

## Abstract

Women with a history of preeclampsia (hxPE) have a ≥4-fold risk for developing cardiovascular disease (CVD) compared with women who had a healthy pregnancy (hxHC). HxPE have exaggerated vasoconstrictor sensitivity to angiotensin (ang) II after pregnancy, which likely contributes to CVD progression after preeclampsia. Ang II-mediated constriction via ang II type 1 receptors (AT1R) is countered by vasodilatory ang II type 2 receptors (AT_2_R); however, the extent to which reductions in AT_2_R-mediated responses contribute to exaggerated ang II-mediated constriction after preeclampsia remains unknown. We examined the balance of AT_1_R- and AT_2_R-mediated responses in hxPE and hxHC (*n*=12/group). We hypothesized that (1) attenuated AT_2_R-mediated dilation would be improved with AT_1_R inhibition in hxPE, and (2) AT_2_R inhibition would increase ang II-mediated constriction in hxHC but have no effect in hxPE. We measured cutaneous vascular conductance responses to compound 21 (AT_2_R agonist; 10^-14^–10^-8^mol/L) alone or with losartan (AT_1_R antagonist; 43 μmol/L) to assess AT_2_R-mediated dilation, and ang II (10^−20^–10^−4^ mol/L) alone or with PD-123319 (AT_2_R antagonist; 1 μmol/L) to assess the role of AT_2_R in vasoconstrictor sensitivity to ang II. Reduced AT_2_R-mediated dilation in hxPE (*P*=0.002) was improved with AT_1_R inhibition (*P*<0.001). Vasoconstrictor sensitivity to ang II was greater in hxPE compared with hxHC (*P*<0.001). Circulating AT_1_R agonistic autoantibodies (AT1-AA) were elevated in hxPE (*P*=0.015). AT_2_R inhibition increased the vasoconstrictor response to ang II in hxHC (*P*<0.001) but had no effect in hxPE (*P*=0.19). These data suggest that hxPE has reduced AT_2_R-mediated dilation that contributes to increased ang II vasoconstrictor sensitivity after preeclampsia.

## Introduction

Preeclampsia is defined as new onset hypertension in pregnancy accompanied by end organ dysfunction [1]. Although the clinical preeclampsia symptoms resolve following delivery, women with a history of preeclampsia (hxPE) have a significantly greater risk of cardiovascular disease (CVD) morbidity and mortality than women who had normotensive pregnancy [2-4]. Vascular studies reveal that women with a hxPE have subclinical vascular dysfunction that likely contributes to the elevated CVD risk [5-8]. Given that CVD is the leading cause of death among women worldwide, healthy women with a hxPE represent a high-risk cohort that requires early mechanism-specific intervention to prevent or delay CVD development.

One putative mechanism underlying vascular dysfunction after preeclampsia is dysregulated renin-angiotensin-aldosterone system (RAAS) signaling, including an increased vasoconstrictor sensitivity to angiotensin (ang) II. Ang II binds to ang II type 1 receptors (AT_1_R) on vascular smooth muscle to cause vasoconstriction or to ang II type 2 receptors (AT_2_R) on the endothelium to induce vasodilation [9]. Women with a hxPE have greater vasoconstrictor sensitivity to ang II, including an exaggerated pressor response [10,11] and increased microvascular constriction [12-14] compared with healthy postpartum controls. This increased vascular sensitivity is found in the absence of differences in circulating ang II [11,15], although AT_1_R agonistic autoantibody (AT1-AA) concentrations, which are elevated during preeclampsia and contribute to a pro-constricted and anti-angiogenic milieu during preeclampsia [16,17], are also elevated postpartum [18-20]. Our group has recently demonstrated that exaggerated ang II-mediated constriction can be reduced in the microvasculature with local or systemic pharmacological inhibition of AT_1_R [13,14], or local activation of the counterregulatory RAAS with ang 1–7 [12] or AT_2_R agonism [15], suggesting that approaches that counteract excessive AT_1_R activation may be feasible strategies to improve vascular function in women who had preeclampsia prior to the onset of chronic CVD.

AT_2_R is an endogenous counterbalance to AT_1_R-mediated responses, and the imbalance of the constrictor AT_1_R and dilatory AT_2_R RAAS axes plays a prominent role in the pathophysiology of preeclampsia. Specifically, the up-regulation of AT_2_R present during healthy pregnancy [21,22] is absent during preeclampsia [23,24] and likely contributes to exaggerated AT_1_R-mediated vasoconstriction postpartum [11]. We have recently demonstrated that women who had preeclampsia have reduced AT_2_R-mediated dilation that contributes to persistent microvascular endothelial dysfunction after preeclampsia [15]; however, no studies have mechanistically examined the role of AT_2_R in exaggerated ang II-mediated constriction in women with a hxPE.

The purpose of the present study was to determine the extent to which changes in AT_2_R signaling contribute to exaggerated activation of the vasoconstrictor RAAS axis via AT_1_R in otherwise healthy women with a hxPE compared with matched control women with a history of healthy pregnancy (hxHC). Our overarching hypothesis is that the balance of AT_1_R:AT_2_R is altered to favor ang II-mediated vasoconstriction in women who had a pregnancy complicated by preeclampsia. Using the cutaneous circulation as a model of global microvascular function [25,26], we hypothesized that hxPE would have reduced AT_2_R-mediated dilation compared with hxHC, and that local AT_1_R inhibition would improve AT_2_R-mediated dilation in hxPE. Additionally, we hypothesized that exaggerated ang II-mediated constriction in hxPE is a result of reductions in AT_2_R signaling, such that local AT_2_R inhibition would increase ang II-mediated constriction in hxHC but have no effect in hxPE.

## Methods

### Ethical approval

The data that support the findings of the present study are available from the corresponding author upon reasonable request. Written and verbal informed consent were obtained before study enrollment in accordance with the World Medical Association Declaration of Helsinki. All experimental protocols were approved by the University of Iowa Institutional Review Board (IRB no. 202309383) and a U.S. Food and Drug Administration (IND no. 124294) was obtained for the use of the pharmacological agents with intradermal microdialysis.

### Participants

Twenty-four healthy normotensive women who were within 5 years postpartum were enrolled. This included 12 women who had preeclampsia in their most recent pregnancy, diagnosed as new onset hypertension and multi-system organ dysfunction after the 20th weeks of pregnancy by their obstetrician and confirmed by medical chart review [1] (hxPE), and 12 women who had a hxHC.

Participants were recruited via: public advertisement in Iowa City, IA and surrounding communities; the University of Iowa and University of Iowa Health Care Medical Centers mass email; and by contacting eligible participants who had previously consented to being contacted for future research studies within our laboratory. Eight hxPE and 1 hxHC participated in a previous study in our laboratory [15]. All participants completed a medical screening that included a physical examination, collection of health history, urine pregnancy test, and fasted blood chemistry and lipid profile (University of Iowa Diagnostic Laboratories, Iowa City, IA). All participants were 18–45 years of age, premenopausal, and self-reported to be physically active. Exclusion criteria included current or past cardiovascular, renal, and/or metabolic disease, current use of anti-hypertensive or cholesterol-lowering medications or use of these medications within 2 months of study enrollment, current tobacco/e-cigarette use, history of gestational hypertension or gestational diabetes in any pregnancy, or currently pregnant. Race and ethnicity were self-reported by all participants.

### Microvascular reactivity measures

Participants completed one experimental visit and were asked to fast for 8 hours, withhold caffeine for 12 hours, and refrain from strenuous physical activity and alcohol for 24 hours prior. This is a standardized approach utilized by our laboratory and others to control for external influences on vascular function [14,15,27,28]. All experiments were scheduled in the morning, with a start time between 7:00 and 10:00 am local time. Following 5 minutes of local ice application to anesthetize the skin, four intradermal microdialysis fibers (CMA 31 Linear Microdialysis Probe, CMA Microdialysis, Holliston, MA) separated by ≥4 cm were aseptically placed in the left ventral forearm for the local delivery of pharmacological agents. Pharmacological agents were weighed just prior to use, mixed with lactated Ringer’s solution, sterilized with syringe microfilters (Acrodisc; Pall, Ann Arbor, MI), wrapped in foil to prevent photodegradation, and perfused through each fiber at a rate of 2 μL/min (Bee Hive controller and Baby Bee microinfusion pumps; Bioanalytical Systems). Laser-Doppler flowmetry probes were placed in local heaters (Moor Instruments, Wilmington, DE) set to thermoneutral (33°C) directly over each microdialysis site to continuously measure cutaneous red cell flux within the tissue (~1 mm^3^) treated by the microdialysis perfusates. Automated brachial blood pressure and heart rate (SureSigns VS2+, Philips Healthcare, Andover, MA) were measured every 5 minutes throughout the protocol. Blood pressure was measured in the contralateral (right) arm at heart level. Participants were instrumented in a semi-recumbent position and remained in this position for the duration of the visit. Following an initial hyperemia-resolution period (~60 minutes), baseline measurements were collected (~10 minutes), and two separate dose–response protocols commenced as described below.

*Compound 21 dose–response*: Two microdialysis fibers were randomly selected to receive either lactated Ringer’s (control) or 43 µmol/L losartan (United States Pharmacopeial) for the inhibition of AT_1_R [13,15,29]. Following baseline measurements, ascending concentrations of compound 21 (C21; AT_2_R agonist, 10^-14^–10^-8^ M; Sigma-Aldrich) mixed with each site-specific treatment were perfused sequentially for 5 min each to ensure a plateau [15,29]. Following C21 doses, 28 mM sodium nitroprusside (SNP; United States Pharmacopeial) was perfused at 4 μL/min and local heaters were increased to 43°C until a maximal blood flow plateau on each site was obtained (~20 minutes).

*Ang II dose–response*: Two microdialysis fibers were randomly assigned for the local delivery of lactated Ringer’s (control) or 1 µmol/L PD-123319 (Tocris, Ellisville, MO) for inhibition of AT_2_R [30,31]. After baseline was achieved, both sites received ascending concentrations of ang II (10^-20^–10^-4^ mol/L; Tocris), perfused sequentially for 5 min each, mixed with the site-specific treatment [12,13].

### Circulating AT1-AAs

On the day of the experiment, blood samples were collected via venipuncture for the analysis of serum AT1-AA. Sera samples were isolated and frozen (–80°C) until analysis. Circulating AT1-AA was measured using a cardiomyocyte bioassay as previously described in Refs. [32-35]. Total IgG fraction was isolated from serum using a Protein G HP Column (Cytiva, Marlborough, MA) following manufacturer’s instructions. Neonatal ventricular cardiomyocytes were isolated and cultured as previously described in Booz and Baker [36]. Cells were loaded with calcium-sensitive Fluo-4 AM dye (Fisher, Waltham, MA), and the cell beating rate was measured by kinetic fluorescent microscopy. Baseline beating rate was calculated, and sample IgG fractions were applied to the cells. A duplicate of each sample was premixed with specific AT1-AA inhibitory peptide ‘n7AAc’ to confirm specific action of AT1-AA. The change in the beats per minute (ΔBPM) was calculated for each sample and indicates circulating AT1-AA activity.

### Data and statistical analysis

All data collection and analysis procedures were standardized prior to testing. Blood flow data were recorded at 40 Hz and stored for offline analysis (PowerLab and LabChart; AD Instruments, Sydney, Australia). Absolute cutaneous vascular conductance (CVC) was calculated (CVC = laser-Doppler flux/mean arterial pressure) and normalized to either a percentage of site-specific maximum (%max) for vasodilation data or site-specific baseline (%base) for vasoconstriction data. Total individual dilation was calculated as the area under the curve (AUC) from %max across all compound 21 doses between the control and losartan-treated sites. Because ang II can elicit vasodilation at low doses systemically [37] and in the microvasculature [31], net AUC was calculated as the difference between the area above and below baseline (net AUC = AUC above the baseline – AUC below the baseline) to assess individual responses [38] (GraphPad Prism 10.2.2, San Diego, CA). The relation between microvascular responses and circulating AT1-AA was evaluated using linear regression (GraphPad Prism).

Sample size was determined *a priori* by power analysis [repeated-measures analysis of variance (ANOVA); power = 0.80, *α* = 0.05]. Using previously published data with similar primary outcomes [13,15,31] and pilot data, we determined that *n* = 12/group would provide ≥80% power to detect meaningful physiological differences of 15% max for vasodilation sites and 20% base for vasoconstriction sites between groups and across treatment sites within group. Student’s unpaired *t*-tests were used to compare participant characteristics and AT1-AA activity. Paired *t*-tests were used to assess within-group changes in AUC. Dose–response CVC data were analyzed using a three-way repeated measures ANOVA (group*dose*pharmacological site; SAS 9.4, Cary, IN) with post hoc Tukey corrections applied for specific planned comparisons when appropriate. Values are mean ± standard error, and individual values are presented within each figure when appropriate.

## Results

Participant characteristics are presented in Table 1. There were no group differences in age, parity, time postpartum, body mass index, blood pressure, or blood chemistry. Four hxHC (2 oral pills, 1 intrauterine device, and 1 implant) and nine hxPE (2 oral pills, 5 intrauterine devices, and 2 implants) were using a form of hormonal contraceptive. Four hxPE and four hxHC reported antidepressant use at the time of the study visit. Of these eight, one hxPE and one hxHC reported concurrent use of an anti-anxiolytic. Five hxPE received treatment to manage preeclampsia during pregnancy, and six hxPE required pharmacological management acutely postpartum (Table 1). None of the participants were currently using anti-hypertensive medications at the time of the study visit. There were no group or site differences in baseline or maximal CVC (Table 2; all *P*>0.05).

**Table 1:** Participant characteristics.

	hxHC (*n* = 12)		hxPE (*n* = 12)		
	mean ± SD	(range)	mean ± SD	(range)	*P*-value
Age (years)	34 ± 5	(25–39)	35 ± 5	(26–44)	0.70
Race, *n*; %					
White	11; 83		8; 67		
Black or African American	1; 8		3; 25		
More than one race	1; 8		1; 8		
Ethnicity, *n*; %					
Non-Hispanic or Latino	11; 92		10; 83		
Hispanic or Latino	1; 8		2; 17		
Parity (number)	2 ± 1	(1–4)	2 ± 1	(1–3)	0.46
Time post-partum (months)	27 ± 16	(5–56)	35 ± 17	(12–55)	0.20
MAP (mmHg)	81 ± 5	(72–87)	83 ± 7	(72–96)	0.24
SBP (mmHg)	108 ± 9	(93–125)	113 ± 8	(99–126)	0.16
DBP (mmHg)	67 ± 4	(59–76)	69 ± 7	(59–82)	0.45
BMI (kg·m^-2^)	26.7 ± 5.8	(20.3–36.7)	29.4 ± 5.6	(22.4–44.5)	0.26
Total cholesterol (mg·dl^-1^)	168 ± 45	(123–269)	170 ± 24	(138–217)	0.89
HDL (mg·dl^-1^)	58 ± 11	(47–79)	58 ± 10	(41–80)	0.75
LDL (mg·dl^-1^)	97 ± 37	(65–186)	99 ± 19	(71–133)	0.88
Triglycerides (mg·dl^-1^)	55 ± 24	(29–114)	66 ± 16	(44–86)	0.24
Fasting glucose (mg·dl^-1^)	79 ± 7	(72–93)	78 ± 10	(53–87)	0.79
HbA1c (%)	5.0 ± 0.4	(4.5–5.7)	5.2 ± 0.1	(5.0–5.4)	0.09
Time of preeclampsia diagnosis (n)					
Before 34 weeks	--		3		
At or after 34 weeks	--		7		
Postpartum (<7 days)	--		2		
Preeclampsia management during pregnancy (*n*)	--		5		
Low-dose aspirin	--		2		
Calcium-channel blocker	--		2		
Magnesium therapy	--		3		
Acute postpartum treatment (*n*)	--		6		
Calcium-channel blocker	--		5		
β-blocker	--		2		
Magnesium therapy	--		1		

Values are mean ± SD (range). There were no differences in any variable between groups (all *P*>0.05). BMI, body mass index; DBP, diastolic blood pressure; HDL, high-density lipoprotein; hxHC, history of healthy pregnancy; hxPE, history of preeclampsia; LDL, low-density lipoprotein; MAP, mean arterial pressure; SBP, systolic blood pressure. *P*-values determined from Student’s *t*-test.

**Table 2:** Baseline and maximal absolute cutaneous vascular conductance (flux·mmHg^-1^).

Microdialysis site	hxHC	hxPE	Group *P*-value
**Compound 21**			
Lactated Ringer’s			
Baseline	0.3 ± 0.1	0.3 ± 0.1	0.55
Maximum	2.1 ± 1.0	2.4 ± 0.7	0.40
Losartan			
Baseline	0.2 ± 0.1	0.3 ± 0.1	0.82
Maximum	1.8 ± 0.7	1.9 ± 0.7	0.72
**Treatment *P*-value**			
Baseline	0.35	0.19	
Maximum	0.40	0.10	
**Angiotensin II**			
Lactated Ringer’s			
Baseline	0.5 ± 0.5	0.3 ± 0.3	0.38
PD-123319			
Baseline	0.4 ± 0.2	0.5 ± 0.2	0.37
**Treatment *P*-value**	0.23	0.52	

Values are mean ± SD. Cutaneous vascular conductance (CVC) = laser-Doppler flux/mean arterial pressure. Across all variables, there were no group or site differences (three-way RM ANOVA, all *P*>0.05).

### Exaggerated AT_1_R-mediated constriction attenuates AT_2_R-mediated dilation in hxPE

**Figure 1:**
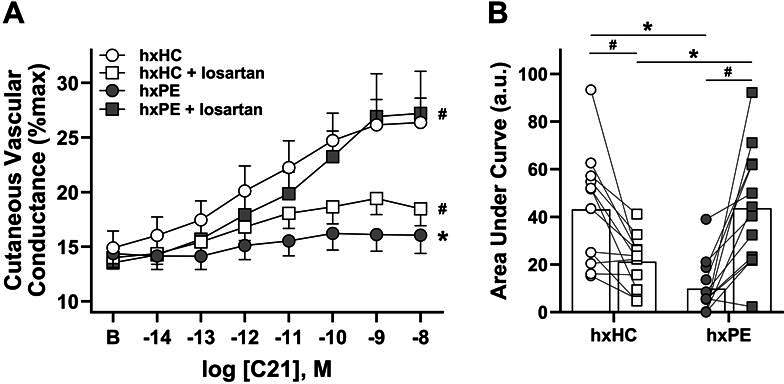
Local losartan treatment improves angiotensin II type 2 receptor (AT_2_R)-mediated dilation in women with a history of preeclampsia. Vasodilation (cutaneous vascular conductance, %max) responses to compound 21 (C21, AT_2_R agonist; **A**) and total individual vasodilation (area under curve, a.u.; **B**) in control (lactated Ringer’s) and angiotensin II type 1 receptor (AT_1_R) inhibited (losartan) microdialysis sites in women with a history of preeclampsia (hxPE, *n*=12) and women with a history of healthy pregnancy (hxHC, *n*=12). *P<0.01 vs. control sites between groups, #*P*<0.01 vs. control site within group. *P*-values determined via three-way repeated measures ANOVA with Tukey post hoc (**A**) or with a Student’s *t*-test (**B**)

Considering the role of AT_2_R during healthy pregnancy and preeclampsia, we first assessed AT_2_R-mediated dilation responses to the specific AT_2_R agonist compound 21 in postpartum women. Microvascular AT_2_R-mediated dilation (CVC, %max) responses to increasing doses of compound 21 were blunted in hxPE compared with hxHC (*P*=0.002, Figure 1A) as was the total vasodilation response (AUC; *P*<0.001, Figure 1B). In order to examine whether AT_1_R-mediated constriction masks AT_2_R-mediated responses in the microvasculature of hxPE, we acutely inhibited AT_1_R with losartan during the assessment of AT_2_R-mediated dilation. Local losartan treatment increased AT_2_R-mediated dilation in hxPE (*P*<0.001 vs. control site) and AUC (*P*<0.001 vs. control site) to values similar to that observed in hxHC (*P*=0.68 hxPE losartan vs. hxHC control). However, in the hxHC group, losartan treatment attenuated AT_2_R-mediated dilation (*P*<0.001) and AUC (*P*=0.005) compared with the control site.

### hxPE have elevated circulating AT1-AA activity, which was negatively associated with AT_2_R-mediated dilation

**Figure 2:**
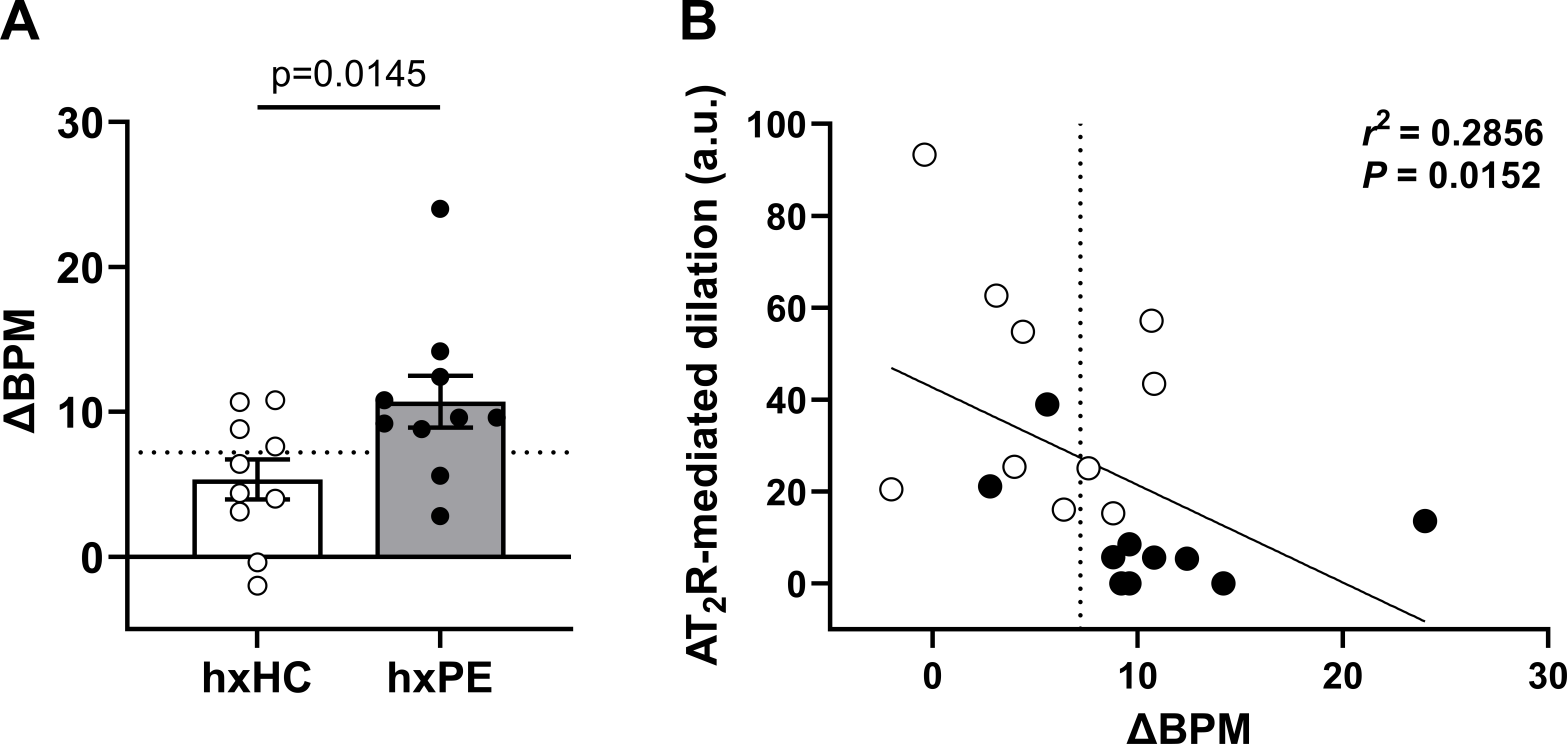
Women with a history of preeclampsia have elevated circulating AT_1_R agonistic autoantibodies that contribute to a pro-constrictor vascular balance. Circulating AT_1_R agonistic autoantibodies (AT1-AA; change in beats per minute, ∆BPM) measured using a cardiomyocyte contraction bioassay (**A**) in women with a history of preeclampsia (hxPE, *n*=10) and women with a history of healthy pregnancy (hxHC, *n*=10). Relation between AT1-AA activity (∆BPM) and AT_2_R-mediated dilation (area under the curve, a.u.) across groups (**B**). Dotted line in both panels denotes cutoff for AT1-AA positive (∆BPM≥7.2) or AT1-AA negative (∆BPM<7.2) values. Statistical comparisons and *P*-values determined by Student’s *t*-test (**A**) and simple linear regression (**B**)

To examine the role that AT1-AA may have in microvascular RAAS responses, we measured activity of circulating AT1-AA and assessed whether AT1-AA activity was associated with microvascular responses in a subset of our participants (*n*=10/group). Eight hxPE and four hxHC had detectable activity of circulating AT1-AA (∆bpm≥7.2). Circulating AT1-AA activity was higher in hxPE compared with hxHC (*P*=0.0145; Figure 2A). Furthermore, circulating AT1-AA activity was negatively associated with AT_2_R-mediated dilation (compound 21 control site AUC; *P*=0.035, *r^2^* =0.22; Figure 2B) across groups.

### Reduced AT_2_R-mediated dilation contributes to exaggerated ang II-mediated constriction in hxPE

**Figure 3:**
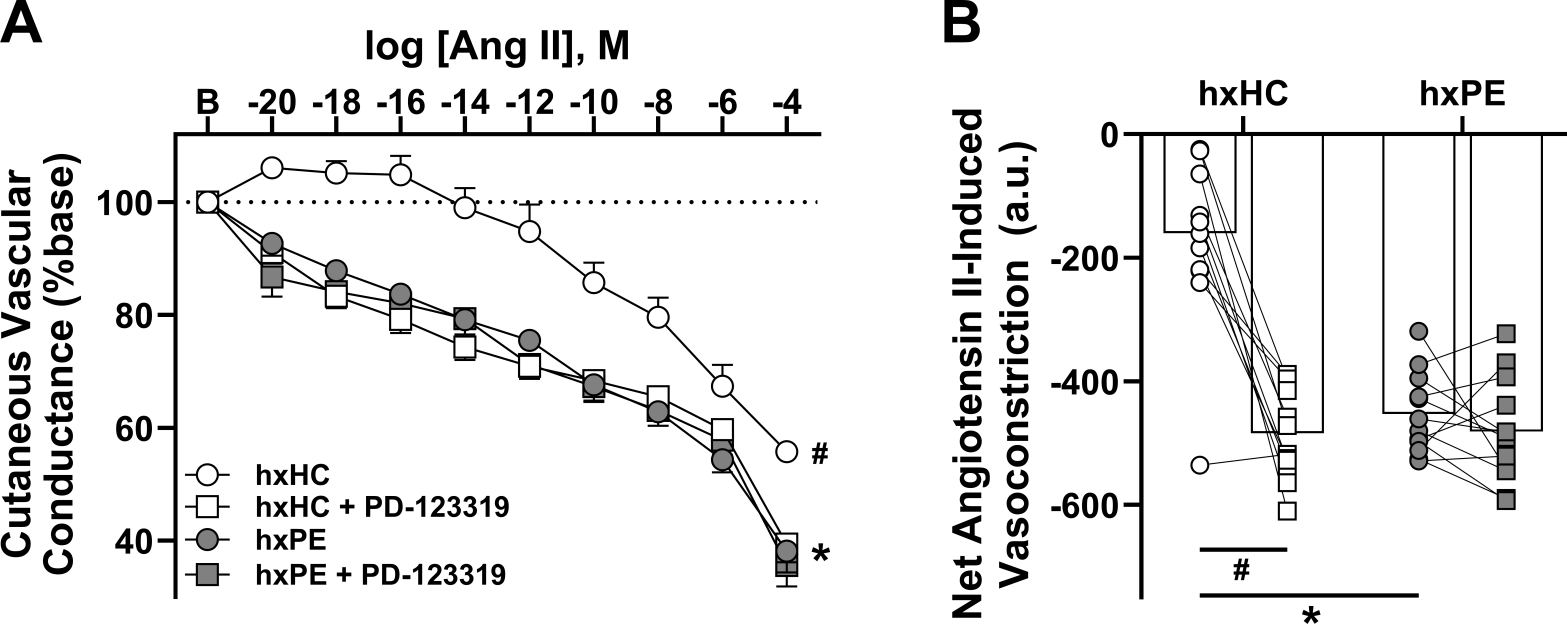
Women with a history of preeclampsia have exaggerated microvascular constriction to angiotensin II mediated by reductions in AT_2_R signaling. Vasoconstriction (cutaneous vascular conductance, %base) responses to angiotensin II (ang II) in control (lactated Ringer’s) and AT_2_R inhibited (PD-123319) microdialysis sites (**A**) in women with a history of preeclampsia (hxPE, *n*=12) and women with a history of healthy pregnancy (hxHC, *n*=12). Net ang II-induced constriction is presented as area under the curve (AUC, a.u.), as the difference between the area above and below baseline (net AUC = AUC above baseline – AUC below baseline; **B**). *P<0.001 vs. control site between groups, #*P*<0.001 vs. PD-123319 site within group. *P*-values determined via three-way repeated measures ANOVA with Tukey post hoc (**A**) or with a Student’s *t*-test (**B**)

Given that women who had preeclampsia have increased vascular sensitivity to ang II, we next examined the role that AT_2_R contributes to this response. hxPE had an exaggerated vasoconstriction (CVC, %base) response to ang II compared with hxHC (*P*<0.001, Figure 3A), and the net vasoconstriction response (AUC) to ang II was greater in the control site in hxPE compared with hxHC (*P*<0.001, Figure 3B). Local AT_2_R blockade with PD-123319 potentiated ang II-mediated vasoconstriction in hxHC (*P*<0.001 vs. control site) but had no effect in hxPE (*P*=0.19) such that there was no difference in PD-123319 treated sites between groups (*P*=0.77) (Figure 3A). Local AT_2_R inhibition increased the net vasoconstrictor response in hxHC (*P*<0.001 vs. control site) but had no effect in hxPE (*P*=0.16) (Figure 3B). There were no group differences in net vasoconstriction in the presence of local AT_2_R inhibition (*P*=0.47). No significant association was found between circulating AT1-AA and ang-II-mediated constriction (ang II control site AUC; all *P*>0.3; data not shown).

## Discussion

The primary findings of the present study are as follows: (1) healthy women with a hxPE have attenuated AT_2_R-mediated vasodilation compared with matched women who had a healthy pregnancy, (2) acute inhibition of AT_1_R with losartan augmented AT_2_R-mediated vasodilation in women with a hxPE, and (3) exaggerated ang II vasoconstrictor sensitivity present in women after preeclampsia is driven, in part, by a reduction in AT_2_R-mediated responses. We also found that women with a hxPE have greater circulating AT1-AA activity than women who had normotensive pregnancy and that there was a negative association between AT1-AA activity and AT_2_R-mediated dilation across groups. Together, these findings suggest that despite the absence of clinical CVD risk factors, healthy women with a hxPE have alterations in the balance of AT_1_R- and AT_2_R-mediated vascular responses that contribute to increased ang II-mediated constriction. This imbalance may contribute to the accelerated progression of CVD following preeclampsia.

Despite the remission of clinical preeclampsia symptoms after delivery, women with a hxPE develop chronic CVD at a younger age (~10 years earlier), with greater frequency, and have higher CVD mortality than women who had a healthy pregnancy [3,4,39,40]. Under physiological conditions, AT_2_R functions not only as an endogenous counterbalance to AT_1_R but also as an independent effector [41,42]. We recently found that AT_2_R-mediated dilation is reduced in otherwise healthy women with a hxPE and that this reduction contributes to endothelial dysfunction in these women [15]. In the current study, we extended these findings and examined the extent to which alterations in the vascular balance of AT_1_R:AT_2_R responses contribute to reduced AT_2_R-mediated dilation in otherwise healthy women with a hxPE. Previous reports demonstrate that in physiological states where the ratio of AT_1_R:AT_2_R expression favors AT_1_R-mediated responses, such as in male rodents [43] and humans [29], AT_2_R-mediated dilation is blunted or even completely masked by tonic AT_1_R activation [44-46]. Considering that women with preeclampsia have increased AT_1_R expression in placental vessels and vascular endothelial cells during pregnancy [23,24,47] and have increased AT_1_R expression in skin biopsy samples after preeclampsia [13], we hypothesized that exaggerated AT_1_R-mediated constriction attenuates AT_2_R-mediated dilation after preeclampsia. Indeed, we found that local AT_1_R inhibition with losartan increased AT_2_R-mediated dilation in hxPE to a magnitude similar to that seen in the control site in hxHC. Given that AT_2_R activation can reduce AT_1_R expression and function [23,48,49], these data support our overarching hypothesis that increased vasoconstrictor sensitivity to ang II after preeclampsia is mediated by alterations in both AT_2_R and AT_1_R signaling. Interestingly, local losartan treatment unexpectedly caused a significant reduction in AT_2_R-mediated vasodilation in hxHC. This finding may be evidence of compound 21 binding to AT_2_R on vascular smooth muscle to produce inflammatory responses in the absence of vascular dysfunction [50,51]. However, examining the role of downstream AT_2_R signaling was not the focus of the present study and requires further investigation.

Circulating AT1-AA activity is elevated and contributes to vascular dysfunction during preeclampsia [17], and circulating AT1-AA remains elevated in some women up to 8 years, following a pregnancy complicated by preeclampsia [18-20]. AT1-AA binds to AT_1_R to elicit AT_1_R-mediated responses that are blocked by AT_1_R inhibitors, and AT_1_R antagonism improves postpartum outcomes in preeclamptic-like rats [52]. In the present study, we found that women with a hxPE had higher activity of circulating AT1-AA than matched control women with a history of normotensive pregnancy. Interestingly, AT1-AA activity was not exclusive to hxPE in our study. AT1-AAs have been detected in patient populations, including hypertension [53,54] and renal-allograft rejection [55], as well as in healthy, normotensive adults [53,54] and in women with a history of healthy pregnancy [19,20]. It currently remains unclear why some apparently healthy adults have detectable circulating AT1-AA activity. We also found that circulating AT1-AA was negatively associated with AT_2_R-mediated dilation, demonstrating that there is a functional role for circulating AT1-AA in reduced microvascular AT_2_R-mediated responses. Furthermore, this association suggests that AT1-AA contributes to a pro-constrictor RAAS balance by masking AT_2_R-mediated responses and increasing ang II vasoconstrictor sensitivity after preeclampsia. However, similar to van der Graaf et al. [20], who demonstrated that the presence of AT1-AA was not correlated with the pressor response to exogenous ang II perfusion in women with a hxPE, we did not find a significant relation between circulating AT1-AA and ang II-mediated constriction in our cohort. It is important to note that AT1-AA non-competitively binds to an allosteric site on AT_1_R that independently activates AT_1_R and increases the sensitivity for ang II to bind [34,56]. Although ang II has greater affinity for AT_2_R than AT_1_R [57], it remains unclear whether or how AT1-AA may affect vascular AT_1_R sensitivity to ang II. While future work specifically examining the effects of AT1-AA on AT_2_R-mediated vascular responses in women who had preeclampsia is warranted, our data suggest that AT1-AA tonically activating AT_1_R to increase ang II vasoconstrictor sensitivity may be one mechanism contributing to AT_2_R dysfunction after preeclampsia.

Although systemic RAAS activity increases during healthy pregnancy, there is a concomitant decrease in vascular ang II sensitivity, resulting in a reduced pressor response to ang II [58]. Conversely, women with preeclampsia have an exaggerated pressor response to ang II [58,59] despite no elevation, or even a decrease [60], in circulating RAAS components during pregnancy. This vascular phenotype likely remains after delivery, as women who had preeclampsia demonstrate an exaggerated pressor response to ang II [10,11] even in the absence of increased systemic RAAS activity [11,15,61,62]. We have demonstrated that increased vascular sensitivity to ang II is present in the cutaneous microvasculature and contributes to reduced endothelium-dependent dilation in these women [12-15,63,64]. Furthermore, we have shown that endothelial function can be improved with local or systemic pharmacological AT_1_R inhibition [13,14], local ang 1–7 treatment [12], or local AT_2_R agonism with compound 21 [15], suggesting that blocking exaggerated AT_1_R-mediated constriction and activating the counterregulatory RAAS are potential mechanistic approaches to improve endothelial function in these high-risk women before the development of overt CVD. Interestingly, one study has demonstrated that women who had preeclampsia have a significant relation between the ratio of AT_1_R:AT_2_R expression in the skin and the change in blood pressure during systemic ang II infusion [11]. The authors report that this relation was absent in women who had a healthy pregnancy and never pregnant normotensive women, suggesting that differences in the vascular tissue RAAS mediate exaggerated responsiveness to ang II following preeclampsia. In agreement, our data extend these findings and demonstrate that exaggerated ang II-mediated microvascular constriction in women who had preeclampsia is due, in part, to a reduction in counterregulatory AT_2_R-mediated dilation. We found that acute AT_2_R inhibition shifted the ang II vasoconstrictor curve downward and increased constriction in hxHC but had no effect on the constriction response in hxPE. Taken together, our data suggest that reductions in AT_2_R signaling contribute to alterations in the RAAS balance that favors AT_1_R-mediated constriction after preeclampsia.

Recent data suggest that preeclampsia may have two distinct phenotypes, often termed ‘early-onset’ (diagnosed before 34 weeks gestation) and ‘late-onset’ (diagnosed at or after 34 weeks gestation) preeclampsia [65]. Early-onset preeclampsia is associated with a greater lifetime risk of adverse vascular outcomes compared with late-onset preeclampsia [66,67]. We did not detect differences in microvascular function when we stratified our data by early- vs. late-onset preeclampsia; however, we are underpowered to make this comparison. Future work is required to determine if differences in microvascular function exist between early- and late-onset preeclampsia subtypes in the years following pregnancy. Similarly, it is likely that the time since the index pregnancy may influence vascular function. Women with a hxPE have premature development of hypertension andCVD, resulting in an increased use of blood pressure- and lipid-lowering medication at a younger age [68]. Therefore, we choose to enroll women up to 5 years postpartum, prior to the manifestation and pharmacological management of clinical disease. Although recent findings suggest that pre-clinical vascular dysfunction in women who had preeclampsia may be reversible at any point up to the development of clinical disease [69], future work examining microvascular function beyond 5 years postpartum in women who did and did not have preeclampsia is warranted.

## Limitations

Our approach did not measure circulating RAAS components in our participants. We and others have demonstrated that systemic RAAS components and their activity are not different in postpartum women who did or did not have preeclampsia [11,15,61,62], suggesting that alterations in ang II receptor sensitivity within the vasculature are the likely mechanism underlying enhanced ang II constrictor sensitivity in women with a hxPE. Another limitation is that we did not control for menstrual cycle phase or contraceptive use, nor did we assess circulating hormone concentrations on the day of the experimental visit. Previous reports suggest that microvascular function is not influenced by menstrual cycle phase [70]. However, the RAAS can be influenced by chronic sex hormone exposure, specifically by differences in estradiol [71-73] compared with testosterone [74,75]. It is also possible that differences in synthetic hormone status based on contraceptive use may have influenced our results. Nine women with a hxPE were utilizing a form of hormonal contraceptive compared with four hxHC women. As such, variations in the number and type of contraceptives (progesterone alone vs. progesterone + estradiol) within each group may have contributed to the variability in our data. Future work designed to specifically assess these outcomes is required to examine how differences in ovarian hormone status and type of contraceptive use may influence mechanisms mediating microvascular responses.

## Conclusions

**Figure 4:**
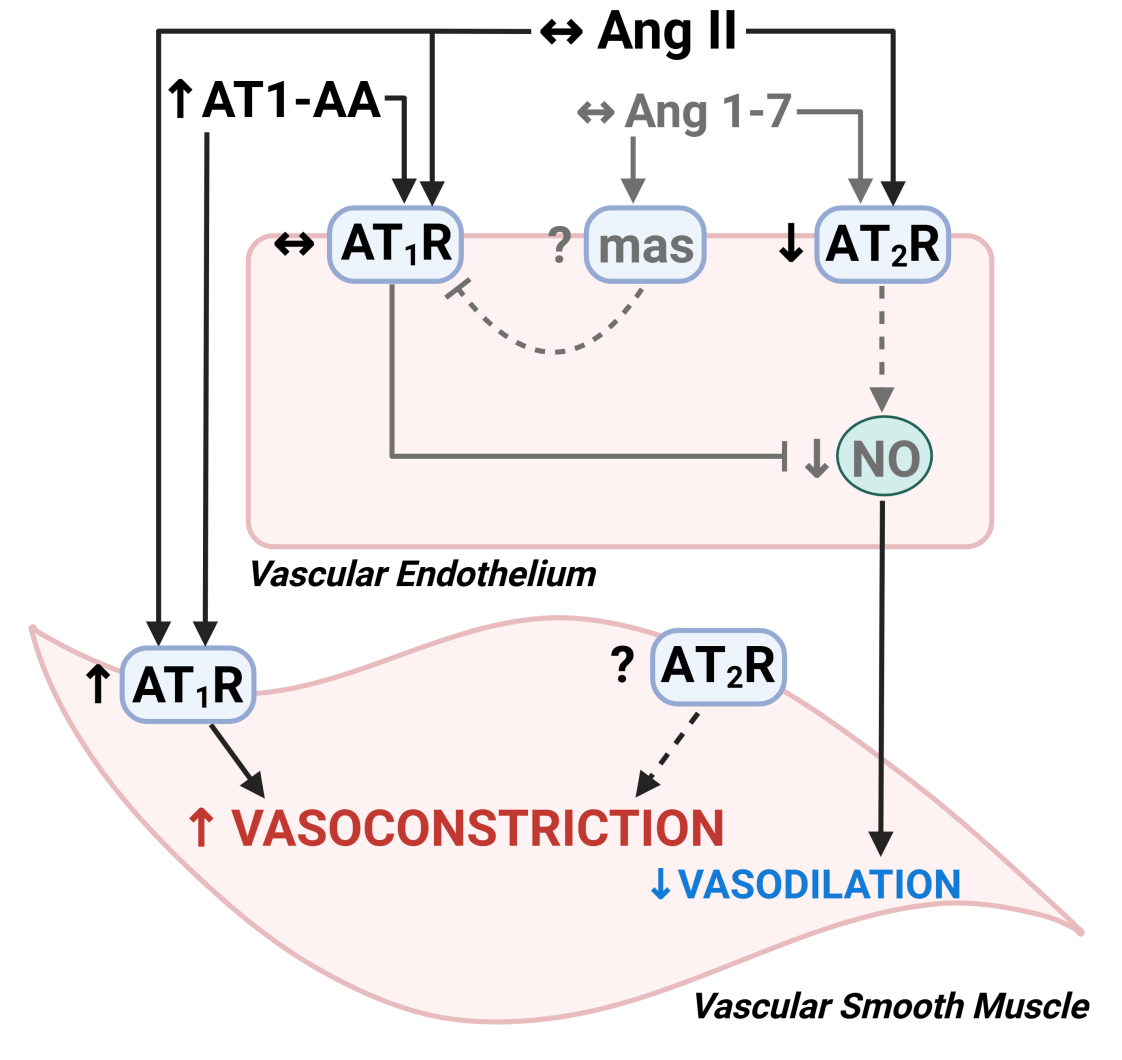
Summary figure of the mechanisms contributing to microvascular dysfunction induced by dysregulated angiotensin (ang) II signaling in women with a history of preeclampsia. An increase in ang II vasoconstrictor sensitivity is mediated by (1) an increase in ang II type 1 receptor (AT_1_R) sensitivity and overstimulation by circulating AT_1_R agonistic autoantibodies (AT1-AA), and (2) a reduction in endothelial ang II type 2 receptor (AT_2_R) expression and AT_2_R-mediated vasodilation. Data presented is a summary of past (gray) and present (black) findings from our group examining ang II-mediated microvascular dysfunction in women with a history of preeclampsia [12-15]. NO, nitric oxide.

We utilized the human cutaneous microvascular bed to examine mechanisms of microvascular function and dysfunction *in vivo*. There is a significant relation between microvascular function measured in the skin and that measured invasively in the coronary and renal microvasculature [26,76], and attenuations in microvascular responses are a direct predictor of CVD morbidity and mortality [77]. Given that systemic microvascular dysfunction drives the clinical presentation of preeclampsia [78,79], it is likely that subclinical dysfunction persists in the maternal microvasculature following delivery [5]. In support of this hypothesis, our group and others have consistently demonstrated that women who had preeclampsia have reductions in microvascular function [8,14,15,63,64,80-82]. We have shown that blocking the increased vasoconstrictor sensitivity to ang II present in the cutaneous microvasculature of women with a hxPE can improve microvascular function [12-14] and that reductions in AT_2_R-mediated responses contribute to microvascular endothelial dysfunction after preeclampsia [15]. In the current study, we extended those findings and demonstrated that reductions in AT_2_R-mediated responses contribute to exaggerated AT_1_R-mediated microvascular constriction after preeclampsia (Figure 4). Collectively, our data indicate that ang II vasoconstrictor sensitivity is present in the microvasculature of women who had preeclampsia and may be mediated, in part, by subclinical alterations in AT_2_R signaling.

**Clinical Perspectives**

- Healthy women with a history of preeclampsia demonstrate an exaggerated vasoconstrictor sensitivity to ang II, due in part to reduced AT_2_R-mediated dilation and elevations in circulating AT1-AA, which likely contributes to the significantly enhanced risk of CVD morbidity and mortality in these women.
- Our data demonstrate that reductions in AT_2_R signaling contribute to exaggerated ang II constrictor sensitivity, resulting in unfavorable alterations in the balance of vascular AT_1_R:AT_2_R function, in women who had preeclampsia.
- Activation of AT_2_R and inhibition of AT_1_R may be a mechanism-specific approach to restore favorable vascular ang II responsiveness and improve microvascular function by reducing exaggerated AT_1_R-mediated vasoconstriction prior to the progression of clinical vascular disease in women who had preeclampsia.

## Data Availability

The data that support the findings of this study are available from the corresponding author upon reasonable request.

## Abbreviations

AT1-AAs: agonistic autoantibodies
AT1R: ang II type 1 receptor
AT2R: ang II type 2 receptor
AUC: area under the curve
BPM: beats per minute
C21: compound 21
CVC: cutaneous vascular conductance
CVD: cardiovascular disease
RAAS: renin-angiotensin-aldosterone system
ang II: angiotensin II
hxHC: history of healthy pregnancy
hxPE: history of preeclampsia
